# Role of iliac crest tangent in correct numbering of lumbosacral transitional vertebrae

**DOI:** 10.3906/sag-1807-258

**Published:** 2019-02-11

**Authors:** Nesrin GÜNDÜZ, Gülçin DURUKAN, Mehmet Bilgin ESER, Ahmet ASLAN, Adnan KABAALİOĞLU

**Affiliations:** 1 Department of Radiology, Göztepe Training and Research Hospital, İstanbul Medeniyet University, İstanbul Turkey

**Keywords:** Computed tomography, iliac crest tangent, lumbosacral transitional vertebra

## Abstract

**Background/aim:**

The iliac crest tangent (ICT) has recently emerged as a reliable landmark to correctly number the lumbosacral transitional vertebrae (LSTV). We retrospectively evaluated the reproducibility and accuracy of the ICT as a landmark in subjects without disc degeneration.

**Materials and methods:**

Fifty-eight patients with LSTV [19 female, 41 (26–52) years] and 55 controls without LSTV [23 female, 40 (26–55) years] who had undergone spinal computed tomography were included. The ICT was drawn on the coronal images, with the cursor in the sagittal view set to the posterior ⅓ of the vertebral body located one level above the LSTV. When more than 1.25 vertebral body was counted below the ICT, the LSTV was considered as S1, otherwise it was considered as L5. The gold standard was counting the vertebrae craniocaudally.

**Results:**

The interobserver agreement was good for determining ICT level (Cohen’s kappa = 0.78, P < 0.001). The rate of correct numbering by ICT in the LSTV group was significantly less than in the controls (43.1% vs. 96.4%, respectively, P < 0.001). Patients with sacralization had a significantly lower correct numbering rate than patients with lumbarization (33.3% vs. 63.2%, respectively, P = 0.03).

**Conclusion:**

ICT does not seem to be a reliable landmark for correct numbering of LSTV in patients with no intervertebral disc degeneration.

## 1. Introduction

Lumbosacral transitional vertebrae (LSTV) is a congenital variant of spine segmentation involving the fifth lumbar (L5) and the first sacral (S1) vertebrae (1). It is relatively common and seen in the range of 4% to 36% of the general population (2). Two broad categories of LSTV include sacralization (L5 resembling a sacral vertebra) and lumbarization (S1 resembling a lumbar vertebra). There are 4 lumbar vertebrae in patients with complete sacralization, whereas 6 lumbar vertebrae are present in complete lumbarization (3). However, intermediate variants of LSTV with incomplete transitions exist (4). A commonly used and more detailed categorization system for LSTV is the Castellvi Classification, in which partial or complete and unilateral or bilateral involvement of the transverse processes are taken into account (5). Castellvi type I indicates the presence of dysplastic transverse process, type II defines pseudarthrosis (partial LSTV), type III corresponds to osseous fusion (complete LSTV), and type IV is a combination of type II and III. A further subclassification is made for each type with “a” for unilateral and “b” for bilateral involvement.

Whole spine imaging is the gold standard method for correct numbering of vertebrae. However, in routine daily practice, isolated lumbar vertebral imaging is conducted in most patients with signs and/or symptoms of suspected lower-spine pathology. Patients with LSTV are rarely symptomatic (e.g., Bertolotti’s syndrome, low back pain) (6,7) and Castellvi types I and IIA can easily be overlooked if only sagittal scans are examined without coronal sections. Clinicoradiological mismatch can be encountered in patients with previously unknown LSTV (8). For instance, in a patient with signs of L5 radiculopathy, the LSTV can be erroneously numbered as S1 in isolated lumbar scanning.

The recognition of LSTV is essential for preinterventional or preoperative assessment of the lumbar spine for accurate surgical planning and avoiding erroneous treatment (3,8,9). Various paraspinal structures have been studied as landmarks for correct numbering of vertebrae including costal facets, aortoiliac bifurcation, inferior vena cava confluence, right renal artery take-off, celiac trunk, superior mesenteric artery origin, iliolumbar ligament, and psoas muscle origins. 

However, previous studies investigating the diagnostic accuracy of these landmarks for correct numbering of LSTV have reported conflicting results (9–14). High rates of anatomic variations of these landmarks and differences between studied patient groups might have contributed to the inconsistencies between the studies and make the usefulness of these landmarks for this purpose debatable. The iliolumbar ligament, in contrast to other landmarks, was once reported to invariably originate from the L5 transverse process (11). 

However, others proposed that the iliolumbar ligament does not universally denote L5 but rather simply depicts the last lumbar vertebra (13,15). Hence, although it has a relatively constant origin, the iliolumbar ligament is also insufficient for correct numbering of LSTV in lumbar scans. The iliac crest tangent (ICT) has recently been reported as an accurate landmark in correct numbering of vertebrae in patients with intervertebral disc degeneration (14). Data regarding the accuracy of the ICT in correct enumeration of LSTV in other patient groups are lacking. In the current case-control study we aimed to evaluate the value of the ICT as a landmark in patients without disc degeneration. 

## 2. Materials and methods

### 2.1. Patient population and study design

We retrospectively assessed the whole spine computerized tomography (CT) images of patients who were scanned for suspected trauma of the spine in the emergency department of our institute between May 2015 and July 2017. A total of 646 patients with whole spine CT were identified. The images were reloaded from the picture archiving communication system (PACS) of our institute. Twelve patients were excluded due to a small field of view not allowing clear demonstration of the uppermost points of the iliac crests. An additional 127 cases were excluded due to significant degenerative disc disease (reduced disc height with or without vacuum phenomenon, reactive end-plate changes, spondylophytes, and sclerosis), scoliosis, vertebral fracture, or severe movement or metallic artifacts. Within the remaining 507 patients, 58 (11.4%) had LSTV according to the Castellvi definition. This constituted the patient group. A total of 55 age- and sex-matched subjects without LSTV were randomly selected as the control group. Institutional ethics committee approval was obtained (decision number 2017/0123). The patients’ age, sex, Castellvi type, level of ICT, estimated number of LSTV provided by ICT analysis, correct number of LSTV provided by counting the vertebrae, vertebral level of aortoiliac bifurcation, right renal artery take-off, and iliolumbar ligament origin were recorded in a database.

### 2.2. Computed tomography data acquisition and interpretation

All patients were scanned in the supine position with the spine longitudinally aligned with the z-axis. The machine was a 16-slice multidetector CT scanner (Optima CT 520, GE Healthcare, USA). A thin-slice (1.25 mm) detector collimation and a low pitch (0.5) with a fixed tube voltage of 120 kV and current of 350 mAs were used. The scan field of view included nonenhanced CT of the head and neck and a contrast-enhanced CT of the thoraco-abdomino-pelvic region as a prerequisite of our institutional CT algorithm in trauma patients. The data acquisition included both soft tissue and bone kernels, and related reconstruction algorithms were used in all patients. In addition to axial, coronal, and sagittal reformations, curved reformats were also used as necessary. Two radiologists with more than 2 years of experience in spine CT imaging who were completely blinded to the patients’ data analyzed the CT images. The readers were required to assess the number of LSTV according to ICT measurement without preassessment of the correct LSTV number. Subsequently, the inconsistencies between the readers were eliminated with consensus decision, which provided the final data to be included in statistical analysis of ICT accuracy. Thereafter, the whole vertebrae were counted down to depict the correct number of LSTV. Finally, all other data of predefined landmarks were also obtained.

**Figure 1:**
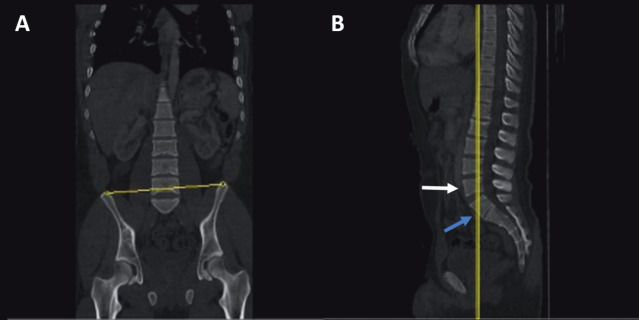
A) Sagittal CT of spine, yellow line crossing the posterior half of the vertebra (white arrow) located one level above LSTV (blue arrow). B) The corresponding coronal CT demonstrating the ICT passing through the uppermost points of the iliac crests. Note the less than 1.25 vertebra, indicating presence of sacralization.

**Figure 2:**
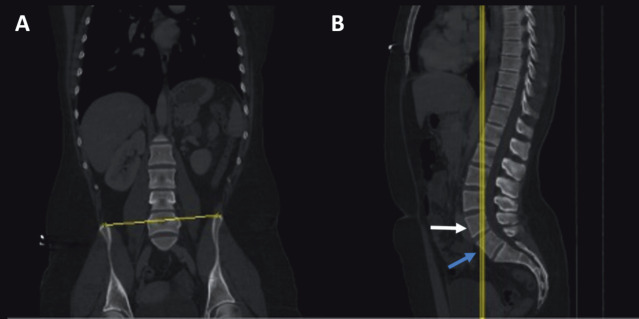
A) Sagittal CT of spine, yellow line crossing the posterior half of the vertebra (white arrow) located one level above LSTV (blue arrow). B) The corresponding coronal CT demonstrating the ICT passing through the uppermost points of the iliac crests. Note the more than 1.25 vertebra, indicating presence of lumbarization.

The ICT was drawn as previously described by Farshad-Amacker et al. (14). A two-window display, one including the coronal CT and the other including the sagittal CT views, was used for the ICT analysis (Figures 1a, 1b, 2a, and 2b). The LSTV was first noted in the sagittal view. The cursor was adjusted to cross the posterior half of the vertebral body adjacent to the LSTV, which is located one level superiorly (Figures 1a and 2a). The corresponding section on the coronal plane was used to draw a line through the uppermost points of the iliac crests (Figures 1b and 2b). This line, the ICT, crossed the lumbar spine. When more than 1.25 vertebral body was counted below the ICT, the LSTV was considered as S1 (lumbarization) (Figure 1b), otherwise it was considered as L5 (sacralization) (Figure 2b). Since LSTV was not present in the control group, the most caudal lumbar vertebra was used instead of LSTV and the penultimate lumbar vertebra was used for ICT drawing and measurement. After obtaining all ICT-related data, the whole vertebrae were counted down to determine the exact number of LSTV.

### 2.3. Statistical analysis

Statistical analysis was performed with SPSS 19.0. The variables were analyzed with the Shapiro–Wilk test to determine whether they were approximately normally distributed. Descriptive statistics were reported as the mean with standard deviation for continuous variables with normal distribution and as the median with 25th–75th percentile values for those without normal distribution. Categorical variables were reported as frequencies with percentages. Patients were first divided into two groups: “Group LSTV” and “Group Controls”. The LSTV Group was further subdivided into “Group Lumbarization” and “Group Sacralization”. Independent two-group comparisons for continuous variables without normal distribution were tested using the Mann–Whitney U test. For continuous variables with a normal data distribution an unpaired t-test was used. The proportions were compared between the groups using Pearson’s chi-square test if the assumptions for the test were met. Otherwise, Fisher’s exact test was used whenever at least one expected count in the contingency table cells was less than 5, and Yates continuity correction was used when less than 25. The interobserver agreement was assessed by Spearman’s test for categorical variables. Significance level was accepted at P < 0.05 for all statistical analyses.

## 3. Results

 Controls 

###  Controls 3.1. LSTV vs. Controls

A total of 113 patients, including 58 with LSTV and 55 age- and sex-matched controls without LSTV, were enrolled in the study. Their baseline characteristics are reported in Table 1. The median age was similar between the LSTV and control groups (40 (26–54) vs. 40 (26–52) years, respectively, P = 0.98). There were no significant sex differences between the groups (32.8% vs. 41.8% females, respectively, P = 0.32). The numbers of patients with LSTV according to Castellvi type are reported in Table 2. Castellvi type 3 was the most frequent LSTV type (35 (60.3%) of 58 patients) and bilateral involvement was present in 37 (63.7%) cases.

**Table 1:** Comparison of baseline characteristics and CT analysis results between groups.

Variable		Control group (n=55)	LSTV group (n=58)	P
Male sex (n, %)		32 (58.2)	39 (67.2)	0.32
Age (years)a		40 (26–56)	40 (26–54)	0.98
ICT level (n, %)	L4 L4–L5 disc L5	16 (29.1) 19 (34.5) 20 (36.3)	39 (67.3) 2 (3.4) 17 (29.3)	<0.001
AIB level (n, %)^b^	L3 L4 + adjacent discs L5	0 (0) 54 (98.2) 1 (1.8)	7 (12.1) 44 (75.8) 7 (12.1)	<0.001
ILL level (n, %)^c^	L4 L5 S1	0 (0) 55 (100) 0 (0)	28 (50) 24 (42.9) 4 (7.1)	<0.001
RRA level (n, %)	T12–L1 disc L1 L1–L2 disc L2	1 (1.8) 35 (63.6) 13 (23.6) 60 (10.9)	11 (19) 29 (50) 7 (12.1) 11 (19)	0.007

^a^ Since the data distribution was not normal, median and 25th and 75th percentile values are reported. The corresponding P-value was obtained by Mann–Whitney U test. Since the 3 × 2 contingency table revealed multiple expected counts less than 5, category unification [(b L4 and adjacent disc vs. non-L4 and adjacent disc) and (c L5 vs. non-L5)] was performed and analyses were repeated for a 2 × 2 table. The corresponding P-values were obtained by Pearson chi-square test. LSTV: Lumbosacral transitional vertebra, ICT: iliac crest tangent, AIB: aortoiliac bifurcation, ILL: iliolumbar ligament, RRA: right renal artery.

**Table 2:** Number of patients in the LSTV group according to Castellvi type.

Castellvi type	(n, %)
1a–1b	4 (6.9)–6 (10.3)
2a–2b	7 (12.1)–4 (6.9)
3a–3b	10 (17.2)–25 (43.1)
4	2 (3.4)

The ICT always crossed the spine from anywhere between the superior endplate of L4 and the inferior endplate of L5. In the LSTV group the ICT crossed the spine more frequently from L4 than in the controls (39 (67.3%) vs. 16 (29.1%), respectively, P < 0.001). The frequencies of the ICT level according to group are reported in Table 1. The rate of correct numbering of the last lumbar vertebra by ICT was significantly lower in the LSTV group than controls (43.1% vs. 96.4%, P < 0.001). When patients with Castellvi type 1 were excluded, the correct numbering rate in the LSTV group decreased slightly (41.7%), which was still significantly lower than in the control group (P < 0.001).

The aortoiliac bifurcation was less commonly at the level of the L4 body or its adjacent intervertebral discs in the LSTV group compared with controls (75.8% vs. 98.2%, respectively, P < 0.001). The axial line passing from the bifurcation never crossed the body of L3 in the control group, whereas it crossed L3 in 7 (12.1%) cases in the LSTV group. The iliolumbar ligament origin was L5 in all (55 (100%)) subjects in the control group, whereas L5 was the origin only in 24 (42.9%) cases in the LSTV group (P < 0.001). The most common site of iliolumbar ligament origin was L4 (50%) in the LSTV group. The frequencies of right renal artery take-off level were quite variable in the whole study population and differed significantly between the groups (Table 1).

###  sacralization 3.2. Lumbarization vs. sacralization

The LSTV was numbered as S1 in 38 (65.5%) and as L5 in 20 (34.5%) patients according to the ICT. However, when the whole spine was counted down craniocaudally, the exact numbers of lumbarization (LSTV = S1) and sacralization (LSTV = L5) were 19 (32.8%) and 39 (67.2%), respectively. Among 20 patients enumerated as L5 according to the ICT, only 13 (65%) of them were true L5. Among 38 patients enumerated as S1 according to the ICT, only 12 (31.6%) of them were true S1. There were no differences between patients with lumbarization and sacralization in terms of age (41 (25–52) vs. 40 (31–58), respectively, P = 0.97) or sex (63.2% vs. 69.2% males, respectively, P = 0.64). There was a higher rate of correct numbering in patients with lumbarization than sacralization according to ICT (63.2% vs. 33.3%, respectively, P = 0.03). The most commonly crossed vertebra by the ICT was L5 in lumbarization (89.5%) and L4 in sacralization (94.9%) (P < 0.001). 

###  females in LSTV group 3.3. Males vs. females in LSTV group

The rate of correct numbering by ICT in females and males was similar (47.4% vs. 41%, P = 0.65). The prevalence of crossed levels by aortoiliac bifurcation, iliolumbar ligament origin, and right renal artery take-off did not differ between the sexes (P > 0.05 for all).

### 3.4. Interobserver reliability of ICT

For controls, the agreement was 100% between the readers in terms of correct numbering of last lumbar vertebra according to ICT. In the LSTV group, the agreement between the readers was good (kappa = 0.78, P < 0.001). The agreement was good (kappa = 0.68) in those with sacralized L5 and was excellent (kappa = 1, P < 0.001) in those with lumbarized S1.

## 4. Discussion

This study suggests that the ICT is quite reproducible but is not a reliable landmark for correct enumeration of LSTV in patients without degenerative intervertebral disc disease. The ICT sign does not seem to improve the rate of correct numbering of LSTV beyond previously described landmarks. This finding is not affected by sex or age. Our results contradict the previous observation that the ICT can accurately reveal the correct number of LSTV in those with degenerative disc disease (14). The iliolumbar ligament origin, right renal artery take-off, and aortoiliac bifurcation levels were also not useful landmarks for enumeration of LSTV in the current study, which is a replication of previous studies revealing the futility of paraspinal structures in this regard. Whole vertebrae imaging is still the gold standard in correct numbering of LSTV and none of the currently available landmarks seem to replace this method.

The disruption of normal spinal anatomy in patients with LSTV might have contributed to the futility of ICT as a landmark (1). Patients with sacralization were reported to have reduced sagittal dimensions and increased downward slope of pedicles (16). Lumbarization was associated with a shorter distance between facet and promontorium (16). The sacrum has also been reported to align more vertically in patients with LSTV (17). The disc height immediately below the LSTV has been found to be significantly decreased (18). Disc height reduction is even more prominent in patients with bilaterally involved LSTV. The transitional disc is also devoid of typical lordotic alignment (19)., with the lumbar curve more lordotic in those with LSTV (20). These anatomical alterations can affect the number of vertebrae remaining below (i.e. more or less than 1.25 vertebrae) the ICT in patients with LSTV. This may explain, at least in part, how the ICT sign accurately enumerates the last lumbar vertebrae in normal subjects but fails in those with LSTV for this purpose.

The contradiction between the results of our investigation and the previous observation reporting the usefulness of ICT as a reliable landmark is probably caused by the difference between studied samples. While Farshad-Amacker et al. (14) studied patients with disc degeneration, such cases were excluded from our study. We argue that disc degeneration might have neutralized the effects of anatomical alterations caused by LSTV in the spine. The net effect probably resulted in the utility of the ICT sign in correct numbering of LSTV similar to control subjects in that previous study. However, this argument needs confirmation with further research.

One may argue that including patients with Castellvi type 1 in the current study may have confounded the results, since this mild type of LSTV is devoid of clinical significance. However, excluding patients with Castellvi type 1 did not increase the correct enumeration rate in the LSTV group in the current study. We propose that absence of disc degeneration disturbs the utility of ICT as a landmark in patients with any degree of vertebral transition.

Interestingly, the correct numbering rate was notably higher in those with lumbarization than sacralization. The interobserver agreement was excellent in patients with lumbarization but moderate in those with sacralization. One possible mechanism that may explain these observations is the differences in anatomical alterations between the lumbarization and sacralization subgroups. For instance, the facet articulations between the LSTV and the sacrum are typically rudimentary or absent in sacralization. On the other hand, facet joints are frequently observed as osseous fusion in lumbarization. In patients with lumbarization, the disc space between the LSTV and the sacrum is bigger than in sacralization (9). Both LSTV subgroups also have differences in terms of dimensions and slope of pedicles, lamina widths, and the distance between facets and the promontorium. Whether higher diagnostic accuracy of the ICT for correct enumeration of LSTV in those with lumbarization than sacralization is affected by the anatomical differences has yet to be confirmed by further studies.

Although the iliolumbar ligament origin was L5 in all control subjects, this was the case in only 42.9% of the LSTV group. Hence, its use as a landmark for assessment of LSTV number remains problematic, a finding in accordance with previous reports (13). The right renal artery was far from being an accurate landmark for LSTV numbering, since the take-off level was highly variable in both control and LSTV subjects. While the level of aortoiliac bifurcation was relatively constant (L4 or adjacent discs in almost all subjects) in the control group, it was considerably more variable in the LSTV group (beyond L4 and its adjacent discs in ¼ of subjects). This is consistent with previous (10,12) and recent evidence (21).

The major limitations of the current study are the retrospective design and relatively low number of patients included. We also recognize that magnetic resonance imaging would more accurately exclude patients with degenerative disc disease. However, in the current study the patients’ history and CT findings were not suggestive of significant degenerative disc disease. Another drawback may be that the subjects were not patients with back pain who would be, in general, the target population of a low-field lumbar imaging. However, we argue that there is no plausible reason that may preclude extrapolation of our results to LSTV patients with low back pain and no disc degeneration.

In conclusion, although highly reproducible, ICT does not seem to be a reliable landmark for correct numbering of LSTV in patients with no intervertebral disc degeneration. The ICT seems even more futile in patients with sacralization than lumbarization. Other paraspinal structures, including aortoiliac bifurcation, iliolumbar ligament, and right renal artery take-off, are also useless in this regard. Whole spine imaging and craniocaudal counting of vertebrae remain the gold standard for correct enumeration of LSTV.
